# Allometry and Interspecific Differences in the Facial Cranium of Two Closely Related Macaque Species

**DOI:** 10.1155/2011/849751

**Published:** 2011-05-25

**Authors:** Tsuyoshi Ito, Takeshi Nishimura, Masanaru Takai

**Affiliations:** Primate Research Institute, Kyoto University, Inuyama, Aichi 484-8506, Japan

## Abstract

Interpreting evolutionary history of macaque monkeys from fossil evidence is difficult, because their evolutionary fluctuations in body size might have removed or formed important morphological features differently in each lineage. We employed geometric morphometrics to explore allometric trajectories of craniofacial shape in two closely related species, *Macaca fascicularis* and *M. fuscata*. These two species exhibit a single shared allometric trajectory in superoinferior deflection of the anterior face, indicating that the differences in this feature can be explained by size variation. In contrast, two parallel trajectories are demonstrated in craniofacial protrusion, indicating that even if they are comparable in size, *M. fuscata* has a higher and shorter face than *M. fascicularis*. The degree of facial protrusion is most likely a critical feature for phyletic evaluation in the *fascicularis* group. Such analyses in various macaques would help to resolve controversies regarding phyletic interpretations of fossil macaques.

## 1. Introduction

Macaques are medium-sized cercopithecine monkeys, which are currently distributed widely in the southern, southeastern, and eastern regions of Asia, and in a restricted area of northern Africa [1]. It is considered that this group probably arose as early as the Late Miocene in northern Africa and spread to Eurasia by the beginning of the Pliocene [2, 3]. Dispersal to eastern Eurasia occurred by the Late Pliocene, and macaques subsequently accomplished successful adaptive radiation in the southern, southeastern, and eastern regions of Asia [2]. The living species are usually classified into four species-groups, that is, *sylvanus*, *silenus*, *sinica*, and *fascicularis *groups ([2, 4, 5], and see also [6]). 

The *fascicularis *group, which is continuously distributed over a vast area extending in its broadest area from western to eastern Asia, includes four species: *Macaca fascicularis* in the tropical area from Indochina to Indonesia, *M. mulatta* in the subtropical and temperate areas from eastern Afghanistan to China, *M. cyclopis* in the subtropical area of Taiwan, and *M. fuscata* in the subtropical to subfrigid areas of Japan [7, 8]. Evolutionary and dispersal histories of the *fascicularis* group have been proposed based on their morphological variations, fossil records [7, 9], and evidence of the mitochondrial and Y-chromosome genes [10–12]. According to their studies, ancestral *M. fascicularis* might have originated in the equatorial region [7, 9] and subsequently dispersed to the islands on the Sunda Shelf during periods of marine regression due to glacio-eustasy and northward to the mainland of Southeast Asia [7, 9]. *M. mulatta* may have diverged from a stock of  *M. fascicularis* around 2.5 Ma and subsequently, were widely distributed in western, southern, and eastern Asia [10–12]. The eastern populations of *M. mulatta* colonized separately in Taiwan and Japan during the period of marine regression around or before 0.4 Ma and became to *M. cyclopis* and *M. fuscata*, respectively [7, 12]. The eastern populations of *M. mulatta* then retreated southward to its present latitudinal zone during a glacial episode [7, 9, 12]. Thus, the current distribution has been formed by intricate dispersal events of the four species with the recent speciation during the Pleistocene period, during which at least six major glacial episodes occurred with significant climate fluctuations [6, 13].

The four living species of the *fascicularis *group are often discriminated by size. The length of the cranium decreases in following order: *M. fuscata*, *M. cyclopis*, *M. mulatta*, and *M. fascicularis* [7]. Size differences are usually attributed to Bergmann's rule [7], which postulates that animals living in colder regions tend to have larger body sizes than close relatives residing in warmer regions. This adaptation effectively maintains body temperature in colder conditions. In addition to size measurements, the four species are identified by specific anatomical features of the cranium. The lateral orbital rim is relatively thicker, and the orbits, interorbital region, and foramen magnum are relatively narrower in *M. fascicularis* than in the other species. Furthermore, the orbits and neurocranium are relatively wider, and the facial cranium is relatively higher in *M. fuscata* than in the other species [14]. 

Such interspecific differences in cranial morphology could, however, be explained by the differences in body size among the four species, that is, an allometric trend common to the *fascicularis *group. In the studies on Chinese macaques [15], baboons [16], and the entire Papionini tribe [17], such common allometric trends have been commonly observed across species, for example, larger individuals usually have a protruding, inferiorly deflecting face and relatively small orbits as compared to those of the smaller individuals. Because of several intrinsic changes in the distribution and climatic conditions, body sizes have most likely been unstable throughout the evolution of each species of the *fascicularis* group, for example, dental remains of *M. fascicularis* discovered from archaeological sites in Borneo dating back to the Late Pleistocene era are larger than those of the living conspecifics [18]. Such evolutionary fluctuations in the body size might make a fossil specimen look different in some morphological features from its true living relatives, which can be explained by a common allometric trend. Such features reflect climate conditions rather than phylogeny, posing a potential risk of wrongly identifying the phyletic position of a given fossil specimen.

In the present investigation, we employed geometric morphometrics to explore the allometric trajectories of craniofacial shape in *M. fascicularis* and *M. fuscata, *which are quite distinct with respect to morphology, distribution, and climate of habitats. We demonstrated a common allometric trend for the *fascicularis *group, and then, attempted to identify the features, which are independent of size destabilization, in order to differentiate between the two species even in fossil remains.

## 2. Materials and Methods

 The samples used in the present study comprised 645 adult specimens with full eruption of the third molars, including 393 specimens of *M. fascicularis *(151 females and 242 males) and 252 specimens of *M. fuscata *(103 females and 149 males). The specimens are stored in the National Museum of Natural History (Washington, USA), the American Museum of Natural History (New York, USA), the Museum of Comparative Zoology of Harvard University (Cambridge, USA), the Field Museum of Natural History (Chicago, USA), the Museum für Naturkunde of the Humboldt University (Berlin, Germany), the Zoologische Staatssammlung München (Munich, Germany), the Natural History Museum (London, UK), the Hakusan Nature Conservation Center (Hakusan, Japan), and the Primate Research Institute of Kyoto University (Inuyama, Japan).

**Figure 1:**
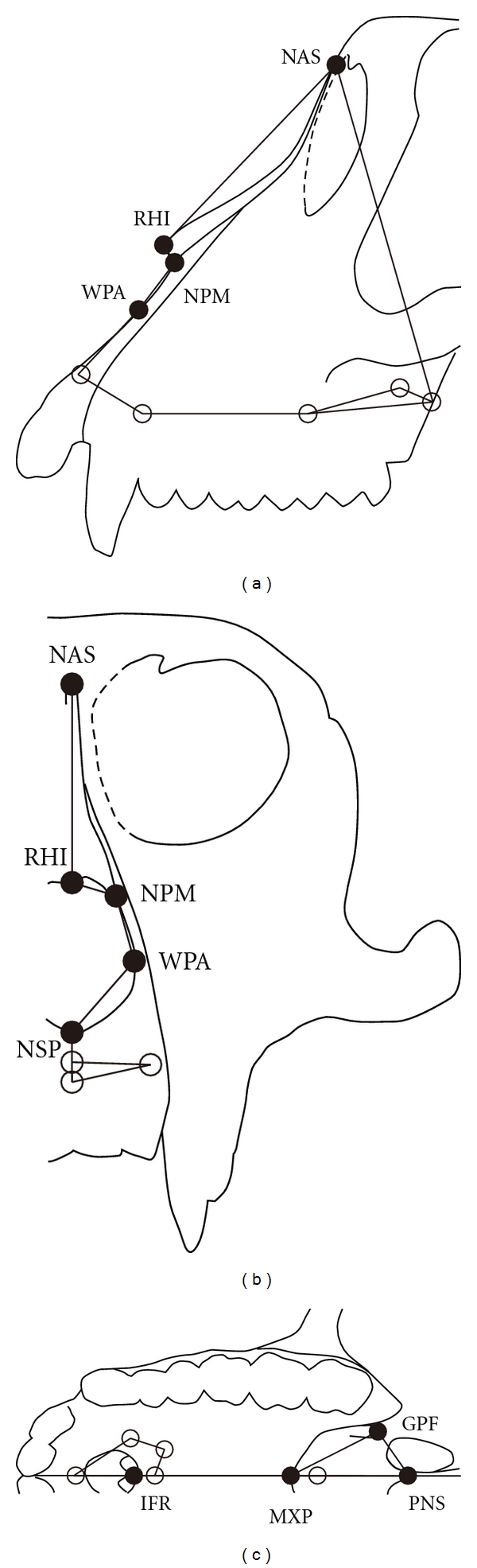
Landmarks and wireframe used in this study (a) Lateral view. (b) Anterior view. (c) Inferior view. The black circle indicates a visible landmark, and the white circle, a hidden landmark.

Three-dimensional (3D) coordinates were measured according to the anatomical landmarks of facial crania by using a 3D digitizer (Microscribe MX; Immersion Corporation, USA). The 9 landmarks measured are shown in Figure 1 and listed in Table 1. The measurements on the left side are typically used for analysis to avoid redundancy. The specimen with the broken left side was analyzed with the measurements of the right side after horizontal reversal. 

The following analyses were performed using the geometric morphometrics software Morphologika version 2.5 [19, 20].

Generalized Procrustes analysis (GPA; [19, 21]) was carried out to register landmark configurations by eliminating the translational and rotational differences and scaling them to the best fit. GPA was achieved by scaling and optimally superimposing all landmark coordinates to minimize the sum of squared distances between homologous landmarks. The registered landmark configurations were then represented as points in the non-Euclidean shape space of Kendall. The centroid size was computed for each specimen at the same time as the square root of the sum of squared Euclidean distances from each landmark to the centroid [19].

Principal component analysis (PCA) was carried out to identify the major axes of variation in the shape of the facial crania. PCA was performed in the tangent plane to Kendall's shape space by using the vector of the tangent space coordinates [19]. 

Shape variability represented by each principal component (PC) was visualized by reconstructing hypothetical forms of the wireframe along each PC. The visualizations were further interpreted using Cartesian transformation grids calculated from the triplets of thin-plate splines (TPS; [19, 22]). The grids derived from TPS indicate the deformation of the space surrounding a reference shape into that surrounding a target shape, wherein the deformation involves minimum bending.

To evaluate our interpretations of shape differences represented by the PCs, we calculated indices and an angular measurement, including Relative Snout Length, Relative Palatal Length, and Facial Deflection on the basis of the digital landmarks (Table 2). Then, correlations between the measurements and the PC scores were examined using R version 2.12.0. [23].

Allometric trajectories for the two species are indicated by PC scores against centroid size, and their differences are examined by analysis of covariance (ANCOVA) and Welch's *t*-test by using R version 2.12.0.

## 3. Results

The centroid size of  *M. fuscata* is significantly larger than that of *M. fascicularis *(*t* = −27.7, *P* <  .001). PC1 and PC2 account for 31.4% and 22.6% of the total variance, respectively, and they are distinct with respect to the PCs enlisted in Table 3. 

**Figure 2:**
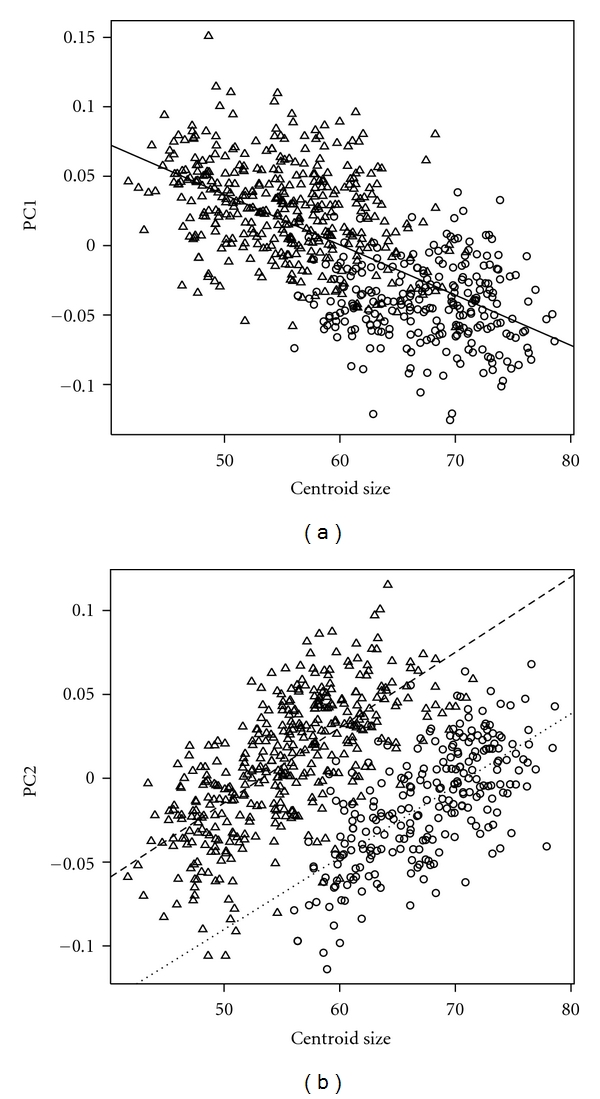
Relation between centroid size and (a) PC1 and (b) PC2. The white triangle represents *M. fascicularis*, and the white circle, *M. fuscata*. The solid line is a regression line for the total samples. The dashed line represents *M. fascicularis*, and the dotted line, *M. fuscata*.

**Figure 3:**
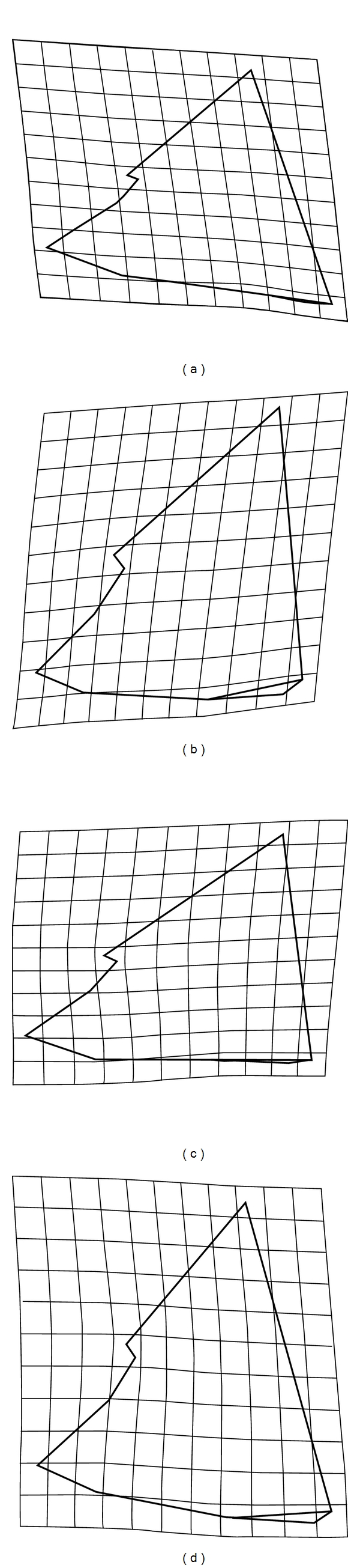
Variations in the craniofacial shapes represented by PC1 (a, b) and PC2 (c, d). Shape differences are visualized by deformation of the wireframe and the transformation grids in the lateral view. Images in the left column (a, c) indicate transformation from the grand mean to the positive extremes in each PC (score = 0.12), whereas the images in the right column (b, d) indicate transformation from the grand mean to negative extremes in each PC (score = −0.12).

PC1 is inversely correlated with the centroid size for all the samples of the two species (*R* ^2^ = 0.42, *P* < .001); *M. fascicularis* samples have significantly higher PC1 score than *M. fuscata* samples (*t* = 26.8, *P* < .001; Figure 2(a)). PC1 indicates anteroposterior shearing of the Cartesian transformation grid, indicating that the anterior portion of the face is deflected more superiorly to produce airorhynchy in a subject with a higher PC1 score (Figure 3(a)), and klinorhynchy in a subject with a lower PC1 score (Figure 3(b)). PC1 is inversely correlated with the angular measurement of Facial Deflection (*r* = −0.88, *P* < .001).

PC2 is significantly correlated with the centroid size separately for each species, *M. fascicularis* (*R* ^2^ = 0.46, *P* < .001) and *M. fuscata* (*R* ^2^ = 0.43, *P* < .001; Figure 2(b)), while *M. fascicularis* has significantly higher PC2 scores than *M. fuscata* (*t* = 8.06, *P* < .001; Figure 2(b)). While the slope is not significantly different between the 2 species (*F* = 0.26, df = 1, *P* = .609), the intercept is significantly higher for *M. fascicularis* than for *M. fuscata* (*t* = 23.9, *P* < .001; Figure 2(b)). PC2 indicates the compression and dilation of the grid, suggesting that the face is lower and longer in the case of higher PC2 scores (Figure 3(c)), and higher and shorter in case of lower PC2 scores (Figure 3(d)). PC2 is significantly correlated with indices of Relative Snout Length (*r* = 0.75, *P* < .001) and Relative Palatal Length (*r* = 0.52, *P* < .001).

## 4. Discussion

The shape variations represented by the first two PCs depend on the size in *M. fascicularis* and *M. fuscata*. These two allometric scaling patterns found in our study are commonly observed across varied clades of the papionin monkeys, for example, in the studies on Chinese macaques [15], baboons [16], and the entire Papionini tribe [17]. However, the two shape variations observed in this study have distinct allometric trajectories with respect to each other.

PC1 exhibits a single allometric trajectory shared by the two species. This suggests that the differences in body size principally produce interspecific differences in the superoinferior deflection of the anterior face between *M. fascicularis* and *M. fuscata*. This indicates that if the hypothetically ancestral *M. fuscata* had been comparable in size to the current *M. fascicularis*, the fossil *M. fuscata* could have a deflected face similar to that of the living *M. fascicularis*. This finding suggests that the deflection in the anterior face should be carefully used for evaluating the phyletic relationships of fossil species to living taxa, at least among the *fascicularis *group.

In contrast, PC2 exhibits two parallel allometric trajectories; the trajectory is vertically lower in *M. fuscata* relative to *M. fascicularis*. This finding means that *M. fuscata* has a higher and shorter face than *M. fascicularis*, even if they are comparable in size. Such a difference is supported by the findings of Fooden [6] and Mouri [24], which demonstrated that *M. fascicularis* has long facial dimensions relative to skull size compared to *M. fuscata* at a comparative age or size. Thus, the differences in the longitudinal-to-vertical proportion of the face against skull size are probably more critical for the phyletic evaluation of a given fossil specimen relative to the living taxa in the *fascicularis *group.

The present findings suggest that for the *fascicularis *group, the degree of facial deflection represented by PC1 likely follows the latitudinal cline in body size, which would lead to klinorhynchy in cold climate. The inferior deflection of the anterior face inevitably extends the nasal profile and cavity; such a feature might have selective advantage for effectively warming the inspired air in the nasal cavity under cold conditions. However, the interspecies differences in this feature per se are rather regarded as simply a consequence of adaptive modifications in body size as predicted by Bergmann's rule.

The variation in facial protrusion represented by PC2 possibly reflects critical modifications occurring during speciation under any selection pressures among the members of the *fascicularis *group. The vertical transposition of the trajectories in the two species could be explained by evolutionary fluctuations in the general scaling. The relatively short face of *M. fuscata *might reflect functional modifications to the masticatory apparatus. Antón [25] also demonstrated such features in *M. fuscata *during the examination of the masticatory apparatus and suggested that the short and high face is desirable for dissipating greater occlusal loads. Indeed, *M. fuscata* may have a tougher diet than the other macaques [26, 27]. On the other hand, Mouri [24] suggested that the long face of  *M. fascicularis* contributes to the large gape, which permitted the presence of long canines in the members of this species. The longer canines of *M. fascicularis*, which could be used as a weapon, are probably related to the severe male-to-male competition within this species as compared to that in *M. fuscata* [24]. Such severe competition may have originated because of the weak seasonality of reproduction by *M. fascicularis*, wherein there are usually only small numbers of females involved in the estrus cycle [14]. Thus, differences in facial protrusions among the members of the *fascicularis *group could be generated by selective processes, which are different for each species, for example, feeding functions and social systems. This would reflect critical morphological modifications occurring during each speciation. 

The macaque fossil specimens from the Pleistocene period found so far are mostly isolated teeth and fragmentary jaws. Nevertheless, several fossil specimens retain facial regions [2, 3, 28], for example, *M. anderssoni* from the Early Pleistocene of Honan, China [29]; *M. robusta* from the Middle Pleistocene of Choukoutien, China [28, 30]; *Macaca* cf. *robusta* from the Middle or Late Pleistocene of  Turupong, South Korea [31]; *M. speciosa subfossilis* from the Late Pleistocene of Thung-Lang, Vietnam [32]; and *M. fuscata* from the Late? Pleistocene of Shikimizu, Japan [33]. The phyletic positions of these specimens are usually evaluated based on geological approximations or morphological similarities to current animals. Nevertheless, fossil specimens occasionally do not possess the stereotypical combinations of cranial features seen in current animals. This tends to confound the process of phyletic reconstruction. For instance, a fossil cranium of *M. speciosa subfossilis* was regarded to be closely related to the extant *M. arctoides* based on geological approximation and morphological similarities in the nasal cavity [34] and malars [35]. However, this specimen does not possess a prominent preorbital concavity, which is a distinct feature of *M. arctoides* [34]. Such ambiguity can be cleared by future studies on allometric trajectories of cranial shape in various macaque subgroups. This approach, as well as the indices and angular measurements correlated with PCs, can be applied to the aforementioned fossil cranial specimens, some of which are facial fragments. Future findings about the allometry and interspecific differences in the dentognathic features will contribute to identifying the phyletic position of more fragmentary fossil materials.

## 5. Conclusions

We demonstrated that two closely related macaque species, *M. fascicularis* and *M. fuscata,* exhibit a single shared allometric trajectory in the supero-inferior deflection of the anterior face, and two parallel trajectories with respect to craniofacial protrusion. The interspecific difference in the latter feature is not explained by the evolutionary modification in body size. The craniofacial protrusion is one of the most reliable characteristics for phyletic evaluation of a given fossil specimen relative to living taxa, at least within the members of the *fascicularis *group. Future efforts with respect to allometric analyses in various macaques are expected to provide critical references for solving continuing controversies about the phyletic relationships between fossil macaques and living taxa.

## Figures and Tables

**Table 1:** Landmarks used in this study.

Abbrev	Definition	Type
IFR	Posterior-most point of incisive foramen	M
MXP	Meeting point of maxilla and palatine along midline	M
GPF	Most posterior point on the margin of greater palatine foramen	B
PNS	Tip of posterior nasal spine	M
NSP	Nasospinale: inferior-most midline point of piriform aperture	M
WPA	Point corresponding to largest width of piriform aperture	B
NPM	Meeting point of nasal and premaxilla on margin of piriform aperture	B
RHI	Rhinion: most anterior midline point on nasals	M
NAS	Nasion: midline point on fronto-nasal suture	M

M: midsagittal; B: bilateral.

**Table 2:** Measurements used in this study.

Variable	Definition
Facial Deflection	Angle between NAS to PNS and IFR to PNS
Relative Palatal Length	Distance from IFR to PNS divided by distance from NAS to PNS
Relative Snout Length	Distance from NAS to NSP divided by distance from NAS to PNS

**Table 3:** Proportion of variance explained by each PC.

	Eigen value	Proportion (%)	Cumulative (%)
PC1	0.00212	31.4	31.4
PC2	0.00153	22.6	54.0
PC3	0.00061	9.0	62.9
PC4	0.00036	5.3	68.2
PC5	0.00034	5.1	73.3
PC6	0.00030	4.4	77.8
PC7	0.00027	4.1	81.8
PC8	0.00026	3.9	85.7
PC9	0.00018	2.6	88.3
PC10	0.00014	2.1	90.4
